# Anchylosed Joints and Other Anomalous Symptoms from Scarlatina

**Published:** 1817-04

**Authors:** 


*For the London Medical and Physical Journal.*

### *Anchylosed Joints and other Anomalous Symptoms from Scarlatina.*

THE ingenious paper of your learned correspondent, Dr. Henning of Zerbst, brought to my mind several peculiarities of Scarlatina, which I do not remember to have seen described in any writer on that disease.

Notwithstanding the industry with which Dr. Willan has collected all the peculiarities of this disease from every author of celebrity, as well as from his own observation, I shall venture to offer a few remarks, which have, I believe, escaped his notice. And first it may not be amiss to say a few words on the paragraph with which he introduces his description of *scarlatina maligna.* "It is truly singular," says he, "that the slightest of all eruptive fevers, and the most violent, the most fatal disease known in this country, should rank together, and spring from the same origin. Experience, however, decides that the simple scarlet fever, the scarlatina anginosa, the scarlatina or angina maligna, and and the scarlet ulcerating sore-throat, without efflorescence on the skin, are merely varieties of one disease. That all of them proceed from the same source of contagion, is evident; because, under the same roof, in large families, some individuals have the disease in one form, some in another, about the same period. According to the state of the air, the soil, climate, or season of the year, one form predominates over all the rest, and gives the general character to every epidemic scarlatina."

Though this last position is generally true, yet it will appear, in a closer sense, inconsistent with the immediately preceding sentence in the same paragraph, where we are told, that, "in large families, under the same roof, some individuals have the disease in one form, some in another, about the same period." The remark is, however, for the most part, just; but not when this ingenious writer speaks of such varieties as peculiar to this contagion.

From the same contagion how numerous are the varieties in the small-pox! In the measles they are scarcely less so. In the prison fever, how numerous are the instances in which members of the court have been alarmingly and even fatally affected, whilst the prisoners have been well enough to attend their trials. In the last instance, indeed, the uniformity has been greater, because the external condition, habits, and the air they have been accustomed to breathe, are different in those who compose the court, and in the inhabitants of the prison; but nothing of this kind is necessary to produce the greatest variety in the exanthemata.

Having premised thus much, I shall proceed to show some varieties in scarlatina, which, if noticed by Willan, have not been described with the minuteness and perspicuity that are usually found in his writings.

The first thing I shall notice may, indeed, be inferred from him, but is by no means so strikingly marked as I have frequently met with it among the patients of the Dispensary. In some of these cases, the whole disease has been confined to the lips, sometimes to the angle of the mouth, from which it sometimes extends to the inside of the cheek. In the last instances, I have seen the ulceration so extensive, and found the fœtor so considerable, that, recollecting the general custom of giving mercury in all cases, I suspected the symptoms to be the effect of a mercurial salivation in a subject peculiarly susceptible of the impression of mercury, and not sufficiently attentive to cleanliness. I was confirmed in this by finding four children in the same family affected in the same manner. These cases proved extremely tedious, and were, throughout, out, attended with extreme debility. However, they all ultimately recovered. The throat in all was little, if at all, affected.

Whilst this family was under my care, two or three others, from the same neighbourhood, were brought to the Dispensary, and one child was unable to leave home. On visiting the latter, I found a severe scarlatina, without any sore throat or sore mouth, excepting that peculiar papulous appearance of the tongue so accurately described by Willan, and so correctly delineated by his engraver. Having now acquired this clue in investigating the cause of the ulcerated mouths, I made no difficulty in ascribing them to scarlatina, and, having hitherto met with them only among the poor, conceived it probable that the children were infected by using the same spoon, which might produce an inoculation, as in the small-pox, and render the superficial part of the disease much more, if not entirely, local, with only a symptomatic fever. But the following history of the progress of the disease in a well-regulated family, of very superior rank, if it does not altogether contradict that theory, at least renders it very doubtful.

The children are three in number. The first had no other symptom but a foul ulcer on the lip, the cause of which was never suspected. It continued to spread, with a sordid bottom and a most disagreeable smell, which no attention to cleanliness would remove or much mitigate. The debility was extreme. In this manner things continued, when a scarlet eruption appeared on another child, extremely mild, but sufficient to excite suspicion and some alarm lest the former should be infected under such unfavourable circumstances. On this subject, however, I had no difficulty in making up my mind that the ulcer on the lip was only scarlatina in a form I had so frequently seen, but chiefly in families very different from the present.

The eruption passed rapidly through its stages, produced only a trifling desquamation, but left the patient extremely reduced in strength. Meanwhile, the third child was seized with the symptoms, and seemed to have passed every danger, when, by its extreme fretfulness, a suspicion was excited that all was not right. At length, a swelling and great tenderness was discovered at each elbow, and afterwards at each knee. The pain and fever were alarming, and the irritability of the child such as almost to preclude the near approach of any one, excepting for the most necessary purposes. In the end, the joints became, to a certain degree, anchylosed, the knees completely so, and the arms admitting a very limited degree of motion. It is the more necessary, that such a case as this should be known, because, though not very infrequent after small-pox, I do not recollect such on event after scarlatina. From its being so little suspected, and from the extreme irritability of the child, sufficient pains could not be used to keep the limbs in the most favourable position for such an altered condition. The arms were, fortunately, in a state of semiflexion, and, in consequence, the little patient has as much use of them as could be acquired; but the knees remain permanently bent and with very little motion. Though this inconvenience, had it been suspected, could not have been entirely prevented, it might to a certain degree, as the upright posture, with a stiff knee, would have been less inconvenient, than the same joint incapable of a full extension.

